# Individualized Responses to Ipsilesional High-Frequency and Contralesional Low-Frequency rTMS in Chronic Stroke: A Pilot Study to Support the Individualization of Neuromodulation for Rehabilitation

**DOI:** 10.3389/fnhum.2020.578127

**Published:** 2020-11-19

**Authors:** John Harvey Kindred, Elizabeth Carr Wonsetler, Charalambos Costas Charalambous, Shraddha Srivastava, Barbara Khalibinzwa Marebwa, Leonardo Bonilha, Steven A. Kautz, Mark G. Bowden

**Affiliations:** ^1^Department of Research and Development, Ralph H. Johnson Veterans Affairs Medical Center, Charleston, SC, United States; ^2^Division of Physical Therapy, College of Health Professions, Medical University of South Carolina, Charleston, SC, United States; ^3^Department of Public Health and Community Medicine, School of Medicine, Tufts University, Boston, MA, United States; ^4^Department of Basic and Clinical Sciences, Medical School, University of Nicosia, Nicosia, Cyprus; ^5^Center for Neuroscience and Integrative Brain Research (CENIBRE), Medical School, University of Nicosia, Nicosia, Cyprus; ^6^Department of Health Sciences and Research, College of Health Professions, Medical University of South Carolina, Charleston, SC, United States; ^7^Department of Neurology, College of Medicine, Medical University of South Carolina, Charleston, SC, United States

**Keywords:** rTMS, rehabilitation, neuromodulation for rehabilitation, NM4R, brain stimulation, corticomotor response, gait rehabilitation, walking speed

## Abstract

**Background**: In this pilot study, we examined the effects of ipsilesional high-frequency rTMS (iHF-rTMS) and contralesional low-frequency rTMS (cLF-rTMS) applied *via* a double-cone coil on neurophysiological and gait variables in patients with chronic stroke.

**Objective/Hypothesis**: To determine the group and individual level effects of two types of stimulation to better individualize neuromodulation for rehabilitation.

**Methods**: Using a randomized, within-subject, double-blind, sham-controlled trial with 14 chronic stroke participants iHF-rTMS and cLF-rTMS were applied *via* a double-cone coil to the tibialis anterior cortical representation. Neurophysiological and gait variables were compared pre-post rTMS.

**Results**: A small effect of cLF-rTMS indicated increased MEP amplitudes (Cohen’s D; cLF-rTMS, *d* = −0.30). Group-level analysis *via* RMANOVA showed no significant group effects of stimulation (*P* > 0.099). However, secondary analyses of individual data showed a high degree of response variability to rTMS. Individual percent changes in resting motor threshold and normalized MEP latency correlated with changes in gait propulsive forces and walking speed (iHF-rTMS, nLAT:Pp, *R* = 0.632 *P* = 0.015; cLF-rTMS, rMT:SSWS, *R* = −0.557, *P* = 0.039; rMT:Pp, *R* = 0.718, *P* = 0.004).

**Conclusions**: Changes in propulsive forces and walking speed were seen in some individuals that showed neurophysiological changes in response to rTMS. The neurological consequences of stroke are heterogeneous making a “one type fits all” approach to neuromodulation for rehabilitation unlikely. This pilot study suggests that an individual’s unique response to rTMS should be considered before the application/selection of neuromodulatory therapies. Before neuromodulatory therapies can be incorporated into standard clinical practice, additional work is needed to identify biomarkers of response and how best to prescribe neuromodulation for rehabilitation for post-stroke gait.

## Introduction

One of the most common impairments after stroke is hemiparesis, i.e., the unilateral loss of muscular strength and coordination of the limb contralateral to the site of the lesion. Lower extremity hemiparesis leads to decreased gait speeds and loss of functional independence (Li et al., 2018). Physical rehabilitation, including physical therapy, is the standard of care for post-stroke mobility and walking impairments. Physical therapy improves walking ability and balance (Pollock et al., 2014), but even with the benefits of physical therapy approximately 26% of chronic stroke survivors over the age of 65 require assistance for activities of daily living, and 30% are unable to walk (Kelly-Hayes et al., 2003). Enhancing the current standard of care could lead to greater physical recovery after stroke, reduce long-term costs of disability, and improve post-stroke quality of life.

Over the last several decades, neuromodulatory techniques have enhanced neuroplasticity through long-term potentiation/depression-like mechanisms (Ma et al., 2014). Repetitive transcranial magnetic stimulation (rTMS) is effective in treating clinical depression (George et al., 1995) and exerts neuromodulatory effects on the corticomotor response (CMR). Measuring the CMR involves applying single or paired TMS pulses to the cortical representation of a target muscle and quantifying the change in the electromyographic signal that follows, known as the motor evoked potential (MEP; Groppa et al., 2012). It is generally accepted when applying rTMS with a figure-of-eight coil to the hand representation in the motor cortex that high-frequency rTMS, ≥5 Hz, excites the corticospinal tract as seen by increases in MEP amplitude (MEP_amp_) elicited in contralateral muscles (Pascual-Leone et al., 1994), while low-frequency, ≤1 Hz, has an inhibitory effect (Chen et al., 1997). This modulation is seen when stimulating the motor cortex directly (Pascual-Leone et al., 1994; Chen et al., 1997) or when applying rTMS to other brain regions that are highly connected to the node of interest, e.g., supplementary motor areas (Matsunaga et al., 2005). When coupled with task-oriented training paradigms, these neuromodulatory effects may be leveraged to improve outcomes through enhanced neuroplasticity in line with the principles of Hebbian learning (Brodie et al., 2014; Ludemann-Podubecka et al., 2016).

The effects of rTMS on the CMR are generally accepted for upper extremity rehabilitation. However, the effects and the potential applications of rTMS for gait and lower extremity rehabilitation are much less researched. Several studies have shown that after multiple sessions of rTMS over the leg motor area similar effects are seen in lower extremity muscles (Wang et al., 2012; Kakuda et al., 2013; Yang et al., 2013; Kim et al., 2015) as in hand muscles. Yang et al. (2013) and Kim et al. (2015) demonstrated in Parkinson’s disease increases in the CMR after delivery of multiple sessions of high-frequency rTMS to the leg motor areas. In individuals post-stroke, the CMR is increased with contralesional low-frequency rTMS (Wang et al., 2012) and after high-frequency rTMS applied bilaterally (Kakuda et al., 2013). Wang et al. (2012) and Kakuda et al. (2013) paired rTMS with task-oriented training and reported improvements in clinical variables such as gait speed, step length, and double-support time. A promising recent meta-analysis by Tung et al. (2019) showed that rTMS paired with lower extremity task-oriented training generally improved clinical outcomes (e.g., LE Fugl-Meyer, gait speed, and Berg balance) to a greater degree than training without rTMS. However, differences in rTMS protocols, task training, and coil types make it hard to form strong conclusions about the effectiveness of physical therapy augmented with rTMS. Additional meta-analyses including rTMS and other types of non-invasive brain stimulations have also stated it is hard to make solid conclusions regarding brain stimulation to enhance gait rehabilitation due to the variability in methodology and reported outcomes (Vaz et al., 2019).

The lack of reported neurophysiological changes in many of the previous studies also does not allow for the elucidation of possible mechanisms of improvement or report on individual response variability. Additionally, due to the high variability in stroke lesion locations, sizes, neural network involvement, and natural adaptive neuroplasticity, it seems unlikely that a single type of rTMS will have the greatest effects on each individual. Fleming et al. (2017) state “Future research should systematically assess differences in response with different stimulation parameters, test measures for who would be most likely to benefit.” To our knowledge, no previous studies have compared low and high-frequency stimulation within an individual to investigate differential responses to various forms of stimulation. Due to the increased size of the magnetic field (Lu and Ueno, 2017) and the location of the lower extremity cortical representations being close to the interhemispheric fissure, perhaps the rTMS applied using a double-cone coil will affect a larger population of corticospinal neurons and interneurons. The increased area of stimulation, that need not be limited to a specific hemisphere, is likely to result in more varied neuromodulation compared to the more precisely applied stimulation with a figure-of-eight coil. These differences are likely exacerbated depending on an individual’s specific neuroanatomy and current nervous system state, suggesting that there is a critical need for studies into individual-level responses to neuromodulation.

The purpose of this pilot investigation was to investigate the group-level effects of ipsilesional high-frequency rTMS (iHF-rTMS) and contralesional low-frequency rTMS (cLF-rTMS) applied with a double-cone coil to the leg motor area on the CMR post-stroke. At the group level, we hypothesized that iHF-rTMS will excite the CMR of the paretic tibialis anterior (TA). We further hypothesized that cLF-rTMS would also result in excitation of the CMR of the paretic TA, possibly due to reduced interhemispheric inhibition originating from the contralesional hemisphere. Additionally, based on previous observations in our laboratory we hypothesized that individual responses would vary to both types of rTMS suggesting that future studies investigating neuromodulation for rehabilitation should be based on individual responses. In a secondary analysis, we investigated if changes in neurophysiological variables in response to rTMS were associated with clinical or neuroanatomical findings.

## Materials and Methods

### Ethical Statement

All methods and procedures were approved by the local Institutional Review Board and conformed to the Declaration of Helsinki. All participants provided signed informed consent before their active participation in the research study.

### Participants and Study Procedures

Twenty prospective participants were recruited from the local Stroke Recovery Research Center and Veterans’ Administration Medical Center patient recruitment databases. Inclusion criteria for the study were: >18 years of age; >6 months post-stroke; Fugl-Meyer lower extremity motor score <34 with preservation of minimal voluntary dorsiflexor and plantar flexor activity; able to walk >10 m; the presence of MEPs in the paretic TA; and able to provide informed consent. Exclusion criteria included: history of seizures; taking medications that could lower seizure threshold; a history of brain injury or other CNS disease other than stroke; the presence of a cardiac pacemaker or other implanted device such as a deep brain stimulator; pre-existing neurological disorders; and severe arthritis or orthopedic conditions that limit the passive range of motion.

**Figure 1:**
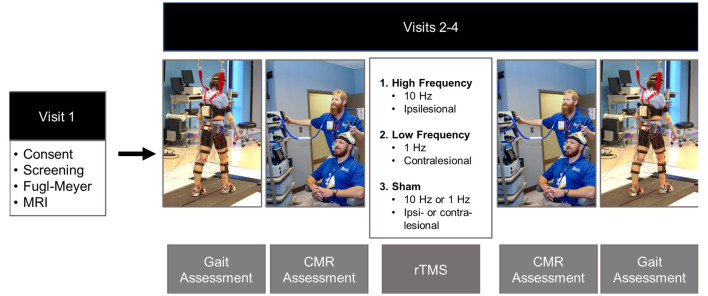
Experimental timeline. Gait and corticomotor response (CMR) assessments were performed before and post rTMS treatments. Gait assessments consisted of over-ground walking on a GAITrite pressure-sensitive walkway and motion capture while participants walked at their comfortable self-selected speed on an instrumented split-belt treadmill. The CMR was tested using single-pulse TMS delivered *via* a double-cone coil to the cortical representation of the paretic tibialis anterior. The rTMS protocol was randomized over Visits 2–4. The protocols included: ipsilesional high-frequency (10 Hz), contralesional low-frequency (1 Hz), and sham. The sham condition was performed by placing a Mu metal insert between the coil and the scalp and randomized to the high-frequency and low-frequency rTMS protocols to maintain blinding of the investigators. Photos are stock photos from our laboratory and consent to use has been obtained.

Once initial eligibility was confirmed *via* phone screening, prospective participants were invited to the laboratory for four visits. During the participants’ first visit they signed informed consent, underwent a structural and diffusion-weighted MRI scan, and a licensed physical therapist performed the Fugl-Meyer LE Motor Assessment (Fugl-Meyer et al., 1975). In subsequent visits, 2–4, participants underwent gait analysis and neuronavigated CMR testing to single-pulse TMS of the paretic tibialis anterior (Figure 1). After initial assessments were performed, participants received 20 min of rTMS. Three types of rTMS were applied in a double-blinded, randomized order: iHF-rTMS, cLF-rTMS, and sham rTMS. Immediately after the rTMS treatments, participants underwent remeasurement of CMR and gait performance. There was a minimum time of 48 h, with an average time of 7 days, between visits to ensure dissipation of neuromodulatory effects that may have occurred from stimulations applied in a previous visit (Touge et al., 2001).

### MRI

After signing informed consent, participants underwent structural MR imaging using a Siemens 3T TIM Trio MRI scanner (Siemens Healthcare, Erlangen, Germany) with a 12 channel head coil. T1 weighted MPRAGE sequence (TR: 1,900 ms, TE: 2.26 ms, TI: 900 ms, Flip angle 9°, acquired isotropic voxel size: 1 mm × 1 mm × 1 mm, SNR: 1), T2-weighted FLAIR (TR: 9,000 ms, TE: 93 ms, TI: 2,500 ms, Flip angle 130° acquired voxel size: 0.9 mm × 0.9 mm × 4 mm, SNR: 1), and Diffusion-weighted images (TR: 6,400 ms, TE: 96 ms, acquired isotropic voxel size: 2.7 mm × 2.7 mm × 2.7 mm, SNR: 1, *b* = 0, 1,000, 2,000 s/mm^2^ for 30 gradient directions) were acquired.

### Gait Biomechanics Assessment

Data acquisition during visits two through four began with gait assessments. Participants walked on an instrumented split-belt treadmill with bilateral force plates (Bertec Corporation, Columbus, OH, USA), Participants performed two treadmill walking trials, each lasting 30 s, walking at their preferred comfortable walking speed. Ground reaction forces (GRF) were sampled at 2,000 Hz. During treadmill walking participants wore a harness connected to the ceiling to mitigate fall risk. Participants did not use any assistive devices, e.g., ankle-foot orthoses, canes, walkers, etc., during treadmill walking nor receive body-weight support from the safety harness. Pre- and post-rTMS assessments were collected at *matched treadmill speeds* for each participant. In addition to treadmill testing, participants walked over a GAITRite pressure-sensitive walkway (CIR Systems Inc., Franklin, NJ, USA) to assess over-ground spatial-temporal variables. Participants performed two trials of over-ground walking on the GAITRite at their self-selected walking speed (SSWS) and their fastest comfortable walking speed (FCWS). Gait measurements were collected before and after rTMS.

### Corticomotor Response (CMR) Testing

**Figure 2:**
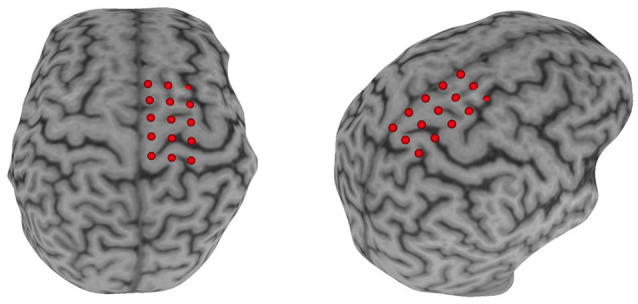
Neuronavigation setup. A Participant’s T1 image was loaded into the neuronavigational software (Brainsight) and a 3 × 5 grid was placed over the M1. The middle row of the grid was placed in line with the motor strip and with the medial column ≈0.5 cm parallel to the interhemispheric fissure.

Surface electromyography (sEMG) was used to record the CMR to single-pulse TMS delivered *via* a double-cone coil powered by two BiStim^2^ stimulators operating in simultaneous discharge mode with an anterior-posterior current direction (The Magstim Company Limited, UK; Sinclair et al., 2006). The CMR was represented by the presence of a measurable MEP (i.e., amplitude >50 μV). An sEMG electrode was placed over the muscle belly of the paretic TA based on published guidelines (Hermens et al., 2000). Before electrode placement, all hair was removed, and the skin was cleaned using alcohol-soaked pads. Once the sEMG electrodes were placed, the participant was seated and registered to the neuronavigation system (Brainsight, Rogue Research, Montreal, QB, CAN) using their structural MRI acquired during their first visit (Charalambous et al., 2018). In the case that a participant’s MRI was unavailable (*N* = 4) we used the averaged MNI brain image native to Brainsight. A 3 × 5 grid, 1 cm distance between grid points, was placed over the ipsilesional motor cortex with the middle row aligned over the motor gyrus and the medial column 0.5 cm lateral to the interhemispheric fissure (Figure 2). All MEPs were recorded in the resting state. If muscle activity was detected before TMS pulse delivery the trial was discarded and repeated. Electromyographic signals were amplified 2,000× (Motion Lab Systems Inc., Baton Rouge, LA, USA) and recorded at 5,000 Hz (Signal 6.03, Cambridge Electronic Design, Cambridge, England, GBR). A single suprathreshold TMS pulse was then applied to each point of the grid on the lesioned hemisphere. This procedure was performed twice, and the recorded MEP_amp_ at each grid point was averaged across the two trials. The grid point with the largest average MEP_amp_ was used as the paretic TA’s “hotspot.” After the hotspot was identified, the resting motor threshold (rMT) was determined using simple adaptive Parameter Estimation by Sequential Testing (PEST; Mishory et al., 2004; Borckardt et al., 2006). Two PEST procedures were performed and the rMT was quantified as the average value of the two measurements. Once the paretic TA’s rMT was determined, 10 single TMS pulses were delivered to the muscle’s hotspot with an interstimulus interval of five to ten seconds, to avoid any neuromodulatory effects, at an intensity of 120% rMT. After 10 MEPs were recorded, participants received 20 min of rTMS. The rMT was reassessed after rTMS application and 10 single TMS pulses were applied using 120% of the post-rTMS rMT.

### rTMS Protocol

The rTMS treatments were randomized and applied using a double-cone coil powered by a Rapid^2^ stimulator (The Magstim Company Limited, UK) within published safety parameters (Rossi et al., 2009). For each type of rTMS protocol, participants received 1,200 single TMS pulses (anterior-posterior current direction, biphasic, single cosine cycle, 400 μs period) delivered at an intensity of 90% of the paretic TA’s rMT. Ipsilesional HF-rTMS was applied to the paretic TA hotspot, lesioned M1, at 10 Hz. Forty trains lasting 3 s each were delivered with an intertrain interval of 27 s. Contralesional LF-rTMS was applied to the contralesional M1 at the grid reference point that mirrored the paretic TA hotspot with an intensity equal to 90% of the paretic TA rMT. This spot was chosen to limit the time participants underwent CMR testing. Contralesional LF-rTMS consisted of single-pulse delivered at 1 Hz for the 20-min treatment period (1,200 stimulations total). Participants immediately underwent remeasurement of the CMR followed by post-intervention gait analysis after the rTMS treatments.

Sham stimulation was accomplished by inserting an MU-metal insert between the TMS coil and the participants’ scalp. The MU-metal insert reduced the strength of the magnetic field and raised the coil from the surface of the scalp increasing the coil to target distance. A custom cloth cover was used during every session to disguise the coil or coil + insert from the investigators performing the rTMS procedures to maintain double-blinding of the experiment. The rTMS set-up was completed by a research coordinator who was not involved in the CMR or gait assessments. This allowed research staff to perform the assessments and applying the rTMS protocol to remain blinded to the rTMS condition. The sham condition was randomized to either iHF-rTMS or cLF-rTMS parameters.

### Data Analyses

#### Gait Analysis

Over-ground walking speed, SSWS, and FCWS were calculated from the GAITRite pressure-sensitive mat. Patients were allowed several feet off the mat at each end, and the first and last footfalls were removed to ensure that starting and ending velocities did not affect the steady-state walking speed over the middle of the mat. Treadmill force plate signals for ground reaction force measurements were sampled at 2,000 Hz. Data were smoothed with a fourth order Savitzky-Golay filter acting on 21 samples and resampled at 100 Hz. Anterior (propulsive) GRFs were used to calculate paretic propulsion (Pp) which shows the propulsive contributions of each leg to the forward movement of the body’s center of mass (Bowden et al., 2006). All post-rTMS kinetic variables were collected at speeds matched to pre-rTMS speeds on the instrumented treadmill.

#### MEP Analysis

Motor evoked potentials were recorded *via* sEMG, no filtering of the signals was performed. Offline analyses of recorded EMG signals were performed in MATLAB R2017b (MathWorks, Natick, MA, USA). Data were imported into MATLAB and demeaned using the average signal over 0.05 s, 0.1 s before and 0.35 s after the TMS trigger pulse. The MEP_amp_ was calculated as the difference between the maximum and minimum values in a 0.08 s window starting at 0.025 s after the trigger pulse. Once amplitude was calculated the signal was rectified and latency, i.e., the time from the TMS trigger pulse to the start of the MEP, was measured. MEP latency was defined as the point when the rectified MEP signal amplitude was greater than the mean plus three standard deviations of the signal amplitude occurring 0.08 s before the TMS trigger pulse (Cacchio et al., 2011; Charalambous et al., 2018; Kindred et al., 2019) for at least 0.001 s. Latency was then normalized to participant height (nLAT) to account for the increased time it takes for the signals to travel along longer neurons in taller individuals and is reported as ms/m. Once all MEP variables were calculated, the data were exported and visually inspected to ensure the accuracy and validity of the values.

#### Image Processing

All images were converted from DICOM to NIfTI format using the dcm2nii tool (Rorden and Brett, 2000). Stroke lesions were manually traced on the T2-weighted images. To track corticospinal tracts, regions of interest (ROI) were manually traced on the Diffusion-weighted images on the pyramidal tract portion of the medulla. Using SPM 12, segmentation of the T1-weighted images into anatomical gray matter regions was performed based on the Atlas of Intrinsic Connectivity of Homotopic Areas (AICHA) brain atlas. The gray matter maps were non-linearly registered into the diffusion imaging space and merged with the manually traced ROIs creating individualized atlases. Tractography was estimated using FSL’s FMRIB’s Diffusion Toolbox (Wang et al., 2007). Tractography streamlines were counted as links between each ROI with each link corrected for the size of ROI and distance between ROIs (Bonilha et al., 2014). To evaluate the connectivity across ROIs, the sum of the streamlines between each ROI was computed. For CST integrity, we computed the connectivity between M1 and pyramidal tract portion of the medulla (M1-CST). We also evaluated the interhemispheric connectivity between M1 of the lesioned and non-lesioned hemispheres (M1-M1) and the intrahemispheric connectivity between SMA and M1 for both hemispheres (M1-SMA). To account for the normal interhemispheric variability, we used the ratio of the streamlines between lesioned and the non-lesioned hemispheres for CST-M1 and M1-SMA (L-N CST = M1-CST_Lesioned_/M1-CST_Non-lesioned_; L-N M1-SMA = M1-SMA_Lesioned_/M1-SMA_Non-lesioned_) shown in Table 1.

**Table 1:** Individual demographic and neuroimaging data.

ID	FM	TSS (months)	Lesioned hemisphere	Lesion volume (mm^3^)	L-N CST ratio	M1-M1 ratio	L-N M1-SMA ratio
1	28	96	L	14,158	0.4	5.0	2.1
2	31	69	L	1,957	0.8	34.9	3.2
3	32	10	R	100,462	0.4	3.2	0.1
4	19	11	L	513	0.1	27.1	1.3
5	24	68	L	76,994	0.2	16.1	2.8
6	31	28	L	69,820	0.8	12.9	3.4
7	27	42	L	1,002	1.7	14.2	1.7
8*	30	72	L	–	–	–	–
9	18	26	L	371	1.5	27.6	3.0
10	24	18	L	237,161	0.0	4.5	2.0
11	25	17	R	1,484	0.6	55.8	1.3
12*	30	9	L	–	–	–	–
13*	19	10	R	–	–	–	–
14*	26	11	L	–	–	–	–

*Images unavailable due to unplanned MRI center shutdown. FM, Fugl-Meyer lower extremity motor test; TSS, time since stroke; L-N, lesioned : non-lesioned; CST, corticospinal tract; SMA, supplementary motor area.

#### Statistics

All data are reported as mean and standard deviation (SD) unless otherwise noted. The distribution of the data was checked using the Kolmogorov–Smirnov test. Data that did not follow a normal distribution were Log_10_ transformed (rMT, MEP_amp_). Comparisons between pre- and post-rTMS variables were performed using two-factor (Treatment: Sham, iHF-rTMS, cLF-rTMS × Time Point: Pre-, Post-rTMS) repeated measures ANOVAs. In the event of significant main effects or interactions, *post hoc* testing was performed using Bonferroni corrections. Cohen’s D measures of effect sizes were calculated using the following formulas:

$$d=\frac{M_{2}-M_{1}}{SD_{\text{pooled}}}\;SD_{\text{pooled}}=\sqrt{\frac{SD_{1}^{2}+SD_{2}^{2}}{2}}$$

We classified effect sizes according to Cohen’s classification of : <0.2 trivial, 0.2–0.5 small, 0.5–0.8 medium, and >0.8 large (Cohen, 1988). Positive effect sizes indicate an increase in values after rTMS while negative effect sizes represented a decrease in post-rTMS variables. To investigate individual responses to the rTMS treatments we performed a secondary analysis investigating if changes in neurophysiological variables with rTMS resulted in clinical changes or if effects were associated with neuroanatomical properties. We calculated percent changes from pre-/post-values as [(X_1__pre −_ X_1__post_)/X_1__pre_] × 100. The distribution of the change scores was assessed using the Kolmogorov–Smirnov test. Pearson’s correlations were calculated between the percent changes with rTMS between CMR variables and gait and neuroimaging variables to investigate if individuals’ changes in CMR were associated with clinical changes after treatment independently. Change scores that were not normality distributed (iHF-rTMS ΔrMT and ΔMEP_amp_) were correlated using Spearman’s rho. The secondary analysis was performed to assist future hypothesis generation and identify possible biomarkers of rTMS response. Due to the preliminary nature of the correlational analysis, no corrections for multiple comparisons were made for secondary analyses. All statistical analyses were performed using SPSS 25 (IBM Corp., New York, NY, USA).

## Results

### Sample Demographics

**Figure 3:**
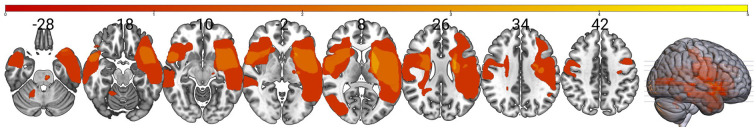
Lesion overlay map for the sample. The map shows an overlay of stroke lesions in standard space indicating a high degree of variability between individuals in the sample (*N* = 10). Color scale indicates the number of stroke survivors having a lesion in every voxel with greater overlay indicated by the yellow. This shows that our sample reflects the highly heterogeneous nature of stroke and prevents using stroke location as a possible response biomarker in this small sample.

We were unable to elicit measurable MEPs in the paretic TA in six participants for a final sample of *N* = 14 (seven Women) even though they maintained some volitional control. The participants’ mean age was 62.6 years (SD 13.4), with a mean time post-stroke of 34.8 months (SD 29.4). The sample was classified as highly functional based on the Fugl–Meyer scores (Lower Extremity Motor, median 27, range 18–32), and the average over-ground walking speed of 0.83 m/s (SD 0.22). Due to an unplanned MRI center shutdown, we were unable to acquire images for four participants. In these cases, neuronavigation was guided using the MNI brain image native to the neuronavigational software (Brainsight). Figure 3 displays a lesion map of the sample showing a heterogeneous sample in terms of areas affected by the lesion.

### Corticomotor Response Variables

#### rMT

**Figure 4:**
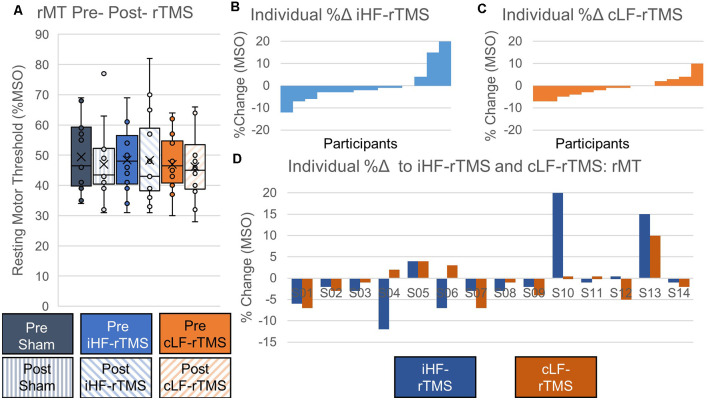
Effects of rTMS on resting motor threshold. The resting motor threshold at the group level is not changed after 20 min of rTMS panel **(A)**. Effect sizes from ipsilesional high-frequency (iHF-rTMS) and contralesional low-frequency (cLF-rTMS) rTMS suggest no effect of rTMS on rMT (Cohen’s *d*, iHF-rTMS, *d* = 0.01; cLF-rTMS, *d* = 0.07). Sham stimulation may result in a slightly lower rMT based on effect size (*d* = 0.21). Individual results, arranged in numerical order from most negative response to most positive response, from ipsilesional high-frequency rTMS panel **(B)** and contralesional low-frequency rTMS panel **(C)** show a high degree of response variability. This suggests that some participants may be more responsive to certain types of stimulation. Panel **(D)** shows each participant’s percent change in response to both types of rTMS.

Data did not follow the normal distribution and were Log_10_ transformed. There were no significant main effects of rTMS on paretic TA rMT at the group level (Treatment, *F* = 0.969, *P* = 0.393; Time Point, *F* = 3.147, *P* = 0.099; Time Point × Treatment, *F* = 0.526, *P* = 0.597; Figure 4A). Independently calculated effect sizes (Cohen’s D, *d*) of both types of rTMS suggest trivial effects on rMT (iHF-rTMS, *d* = −0.01; cLF-rTMS, *d* = −0.07), although there was a small effect of sham stimulation suggesting a decrease in rMT after 20 min of stimulation (*d* = −0.21). Individual responses, reported as %Δ, to iHF-rTMS and cLF-rTMS are displayed in Figures 4B,C. Individual responses to iHF-rTMS and cLF-rTMS ranged from a decrease of 12% to an increase of 20% maximal stimulator output (MSO; Figure 4D).

#### MEP Amplitude

**Figure 5:**
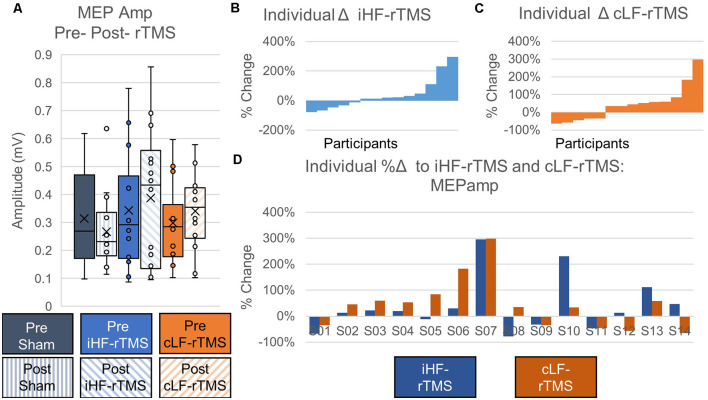
Effects of rTMS on motor evoked potential amplitude. Motor evoked potential amplitude at the group level is not changed after 20 min of rTMS panel **(A)**. Effect sizes calculated from ipsilesional high-frequency (iHF-rTMS) and contralesional low-frequency (cLF-rTMS) indicate slight increases in excitability with both types of rTMS (Cohen’s *d*; iHF-rTMS, *d* = −0.19; cLF-rTMS, *d* = −0.30). Sham stimulation results in a slight inhibition of the MEP_amp_ (*d* = 0.29). Individual results, arranged in numerical order from most negative responses to most positive responses, from iHF-rTMS panel **(B)** and cLF-rTMS panel **(C)** show a high degree of variability in the responses to each type of rTMS. This suggests that some participants may be more responsive to certain types of stimulation. Panel **(D)** shows each participant’s response to both types of rTMS.

Data did not follow the normal distribution and were Log_10_ transformed. There were no significant main effects of rTMS on changing paretic TA MEP_amp_ at the group level (Treatment, *F* = 0.631, *P* = 0.540; Time Point, *F* = 0.036, *P* = 0.853; Time Point × Treatment, *F* = 0.655, *P* = 0.528; Figure 5A). Independently calculated effect sizes were trivial for iHF-rTMS (*d* = 0.19) and small for cLF-rTMS (*d* = 0.30) and indicated an increase in MEP_amp_ with both treatments. Sham stimulation resulted in a small inhibitory effect (*d* = −0.29). Individual responses, reported as %Δ, to iHF-rTMS and cLF-rTMS are displayed in Figures 5B,C. Individual changes in MEP_amp_ in response to iHF-rTMS and cLF-rTMS ranged from −78% to 298% (Figure 5D).

#### Normalized MEP Latency

**Figure 6:**
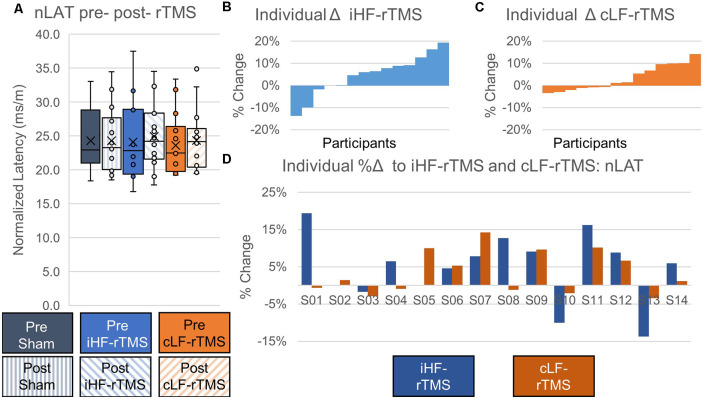
Effects of rTMS on normalized motor evoked potential latency. Normalized latency (nLAT) of the motor evoked potential is not changed after 20 min of rTMS at a group level panel **(A)**. Cohen’s D measures of effect size indicate slight increases in nLAT with ipsilesional high-frequency rTMS (iHF-rTMS, *d* = −0.15) and contralesional low-frequency rTMS (cLF-rTMS, *d* = −0.16). There is no effect from sham stimulation (*d* = −0.03). Individual results, arranged in numerical order from the most negative response to the most positive response, from iHF-rTMS panel **(B)** and cLF-rTMS panel **(C)** show varied responses to both types of stimulation. This suggests that some participants may be more responsive to certain types of rTMS. Panel **(D)** shows each participant’s response to both types of rTMS.

There were no significant main effects of rTMS on nLAT of the paretic TA at the group level (Treatment, *F* = 0.478, *P* = 0.626; Time Point, *F* = 2.738, *P* = 0.122; Time Point × Treatment, *F* = 1.777, *P* = 0.189; Figure 6A). Independently calculated effect sizes of iHF-rTMS and cLF-rTMS indicated a trivial increase in nLAT and no effect of Sham stimulation (iHF-rTMS, *d* = 0.15; cLF-rTMS, *d* = 0.16; Sham, *d* = 0.03). Individual responses, reported as %Δ, are displayed in Figures 6B,C and ranged from −14% to 19% (Figure 6D).

### Gait Biomechanics Assessment

The average SSWS and FCWS did not change with rTMS treatments (SSWS, *F* = 1.014, *P* = 0.377; FCWS, *F* = 0.464, *P* = 0.634) and there were no group effects of rTMS type on SSWS or FCWS (SSWS, *F* = 3.029, *P* = 0.105; FCWS, *F* = 0.163, *P* = 0.693). There were no interactions between the factors for either SSWS or FCWS (*F* < 3.102, *P* > 0.062). The rTMS treatments did not change gait kinetics, measured as Pp (Treatment, *F* = 0.468, *P* = 0.631; Time Point, *F* = 0.000, *P* = 0.992; Treatment × Time Point, *F* = 0.309, *P* = 0.737). Means and SDs for gait variables are listed in Table 2.

**Table 2:** Gait kinetic and spatiotemporal group averages.

	Pre	Post
SSWS (cm/s)—Sham	87.2 (20.4)	93.1 (23.0)
FCWS (cm/s)—Sham	116.9 (30.9)	113.8 (30.5)
Pp—Sham	0.43 (0.14)	0.42 (0.14)
SSWS (cm/s)—HF	92.2 (26.0)	96.7 (23.9)
FCWS (cm/s)—HF	115.7 (32.9)	119.7 (30.7)
Pp—HF	0.44 (0.13)	0.43 (0.11)
SSWS (cm/s)—LF	92.1 (24.3)	93.4 (26.3)
FCWS (cm/s)—LF	117.8 (29.8)	114.2 (29.7)
Pp—LF	0.43 (0.13)	0.44 (0.13)

*No significant differences were detected pre-/post-rTMS. SSWS, self-selected walking speed; FCWS, fastest comfortable walking speed; Pp, Paretic Propulsion; HF, high-frequency; LF, low-frequency*.

### Secondary Analysis of Individual Changes in CMR Variables and Clinical Outcomes

**Figure 7:**
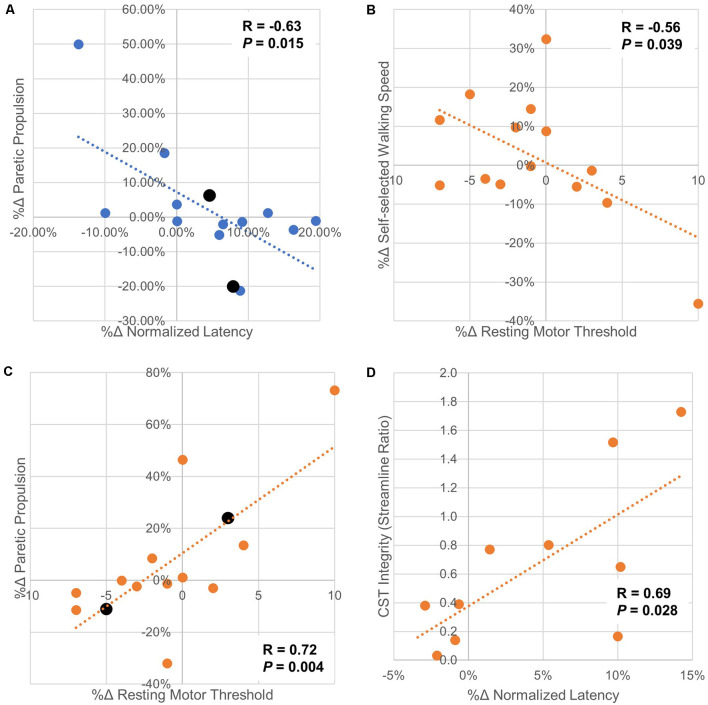
Scatter plots of correlations between changes in corticomotor response and gait/neuroimaging variables. Panel **(A)** Paretic propulsion (Pp) represents the contribution of the paretic side to the forward movement of the center of mass during gait. Symmetry is represented by a Pp of 0.5 with values < 0.5 indicating the impaired/hemiparetic leg is contributing less to forward movement. In this sample, 12/14 participants had a Pp < 0.5. In these cases, a positive increase means a trend toward symmetry. The two participants with Pp > 0.5 are displayed in black and their markers are larger than the other data points. This correlation analysis shows that as normalized latency increased with ipsilesional high-frequency rTMS generally moved Pp further away from symmetry. Panel **(B)** shows that as the resting motor threshold of the paretic tibialis anterior is increased with contralesional low-frequency rTMS, indicating a decrease in excitability, self-selected walking speed also decreases. This may indicate that contralesional low-frequency stimulation should not be used if this stimulation type increased paretic tibialis anterior resting motor threshold as increases in walking speed are generally sought after physical rehabilitation. Panel **(C)** indicated that increases in resting motor threshold with ipsilesional low-frequency rTMS generally increase Pp towards symmetry. It is interesting to note that increases in paretic tibialis anterior resting motor threshold with applied rTMS protocols had opposite effects on changes in Pp. This may indicate that the increase in resting motor threshold is from both types of rTMS are due to differing mechanisms. Panel **(D)** shows that with less connectivity between M1 and the CST in the lesioned side compared to the non-lesioned side there was less change in normalized latency with contralesional low-frequency rTMS. These findings should be interpreted with caution as the study may not have been properly powered for these secondary analyses. However, these data should help guide future hypothesis generation and follow-up experiments.

There were no significant correlations between the percent change in rMT, MEP_amp_, and nLAT and percent change in SSWS or FCWS with iHF-rTMS. However, there was a moderate correlation between the %Δ nLAT and %Δ Pp (*R* = −0.63, *P* = 0.015) suggesting that PP may become more symmetrical if you could decrease nLAT with iHF-rTMS (Figure 7A). The results from cLF-rTMS demonstrate correlations between the %Δ in rMT with SSWS and Pp (SSWS, *R* = −0.56, *P* = 0.039; Pp, *R* = 0.72, *P* 0.004; Figures 7B,C). This indicated increases in rMT of the paretic TA led to slower walking speeds and a greater contribution of propulsion from the non-paretic side. There were also non-significant moderate correlations between the %Δ in rMT and the %Δ in FCWS (*R* = −0.51, *P* = 0.063) and between the %Δ in nLAT and %Δ in FCWS (*R* = 0.48, *P* = 0.083) with cLF-rTMS. These correlations indicated that individuals who had a reduction in paretic TA rMT with cLF-rTMS results in improved gait speeds but the increase may be due to greater non-paretic leg function and not improved function of the paretic leg. Structural connectivity of the CST, expressed as the ratio of streamlines between the lesioned and non-lesioned hemispheres, correlated with the %Δ in nLAT after cLF-rTMS (*N* = 10; %Δ nLAT, *R* = 0.69, *P* = 0.028). There were no other correlations between neuroimaging variables and CMR measures (*R* < 0.58, *P* > 0.078). Significant correlations are plotted in Figure 7.

## Discussion

At the group level, we did not detect significant changes in corticomotor response variables but effect sizes (Cohen’s D) showed that the effects of rTMS treatments on our sample generally had the same direction of change as reported by previous investigators. Our secondary analysis of correlations between %Δ CMR and %Δ clinical gait measures may support the use of neuromodulation of rehabilitation to potentially improve propulsive properties and gait speeds post-stroke, seen if iHF-rTMS resulted in a reduced nLAT there was a change toward symmetry in Pp. These findings support previous studies of physical therapy augmented with rTMS (Wang et al., 2012; Kakuda et al., 2013; Chieffo et al., 2014). Investigations at the individual level also show that some participants responded to a greater degree to one of the two stimulation types and in some cases, the two treatment types had opposite effects. Lastly, we show individuals with more symmetrical CST structural connectivity had less increase %Δ in nLAT of the paretic TA in response to cLF-rTMS, indicating CST connectivity may be a possible biomarker for neuromodulatory responses. These initial results suggest an individualized approach should be considered when providing neuromodulation for rehabilitation with rTMS.

Modulation of the motor cortex representing the lower extremities requires different a methodology compared to typical rTMS delivered to upper extremity cortical representations and frontal lobe locations (Kesar et al., 2018). This is in part due to the location of the lower extremity motor neurons being deeper within the brain and thus needing a more powerful magnetic field to penetrate further from the coil to elicit responses, especially in clinical populations such as stroke. Even with this knowledge, most studies using rTMS to modulate the lower extremity cortical representations have *not* used a double-cone coil (Tung et al., 2019). Along with the few studies measuring clinical outcomes with double-cone coil delivered rTMS post-stroke, there are even fewer studies reporting the neurophysiological changes that occur with this type of treatment (Huang et al., 2018). Huang et al. (2018) showed there were no changes in CMR variables after cLF-rTMS was delivered. However, this study was performed in an acute stroke population so the effects of natural recovery/plasticity on the rTMS treatment are unknown and they dosed their treatment on the active motor threshold of the rectus femoris. Wang et al. (2012) showed increased MEP amplitudes of the rectus femoris on the paretic side with cLF-rTMS post-stroke using a figure-of-eight coil.

There are no direct investigations of iHF-rTMS and cLF-rTMS delivered *via* a double-cone coil on the CMR in the chronic stroke population. The double-cone coil induces a larger deeper penetrating magnetic field (Lu and Ueno, 2017). This larger field will affect a greater number of cells, including targeted CST neurons and interneurons. It should not be expected that the neuromodulatory effects of the figure-of-eight and double-cone coils will be similar. Our results indicate that at the group level the rTMS delivered *via* a double-cone coil may not have large group effects on neurophysiological measures. This suggests to us, that just like in many other rehabilitative protocols, *individualized neuromodulatory therapies should be explored*. Due to the great heterogeneity of stroke characteristics, innate neuroplasticity, and functional recovery post-stroke, it should not be expected that a single rTMS protocol will provide the greatest beneficial modulation of the motor network for each unique patient. Especially when the delivered stimulation is affecting a greater area of the brain and likely includes stimulation to both hemispheres. Previous studies using transcranial direct current stimulation, i.e., tDCS, were affected by lesion location (Rosso et al., 2014), genetic markers (Fridriksson et al., 2018), and gray matter density (Cotelli et al., 2016). These and other unknown factors will likely influence the response to rTMS post-stroke. Participants in some tDCS trials are being classified as up- or down-regulators depending on their response to anodal tDCS. This classification has been shown to explain some of the changes seen in tDCS and stroke rehabilitation studies (Madhavan et al., 2020).

The call to individualize rTMS treatments is supported when we examine the effects of rTMS on the structural connectivity of the CST. We show, albeit in a small sample, that as M1-CST structural connectivity is more symmetrical between the hemispheres there is a lesser increase in nLAT after cLF-rTMS. This suggests that individuals with more symmetric CST connectivity may be more responsive to rTMS delivered to the contralesional side. These findings may also suggest that low-frequency rTMS may interfere with the “normal” functioning of the non-lesioned hemisphere leading to changes that would generally be considered negative in the lesioned hemisphere. Due to the nature of reporting the structural connectivity as a ratio between the lesioned and non-lesioned sides, we are unable to determine if a minimal “threshold” of connectivity is required within the lesioned hemisphere to see the effects of iHF-rTMS. We report structural connectivity as a ratio between lesioned and non-lesioned sides because this allows us to use the non-lesioned side as a pseudo internal control. Similar methods have been used previously in healthy older adults (Bonilha et al., 2014) and post-stroke (Madhavan et al., 2011).

At the individual level, we see that changes in rMT and nLAT correlate with changes in SSWS and Pp. This suggests a possible mechanism between rTMS and some of the clinical improvements reported by other research groups (Fleming et al., 2017; Tung et al., 2019). Although the mechanisms leading to these changes may not be similar between the two stimulation types, ipsilesional stimulation may affect changes in CMR locally while contralesional rTMS changes interhemispheric or other long-distance communications. These changes should be further explored using dual-pulse TMS paradigms which can allow for measurements of intrahemispheric communications. This also highlights the need to measure neurophysiological changes in the lesioned and non-lesioned hemispheres. Clinical changes post rTMS may come in the form of improved function of the paretic leg or by improved compensation strategies performed by the non-paretic leg. One of the limitations of this study, and others, is that most investigations of rTMS and physical rehabilitation post-stroke have had small sample sizes (i.e., <20), making the generalizability of the results difficult. We must also consider that our CMR measures are taken at rest. The resting state of the motor network may not reflect the state of the network during task performance, and it may be more appropriate to determine how rTMS affects the CMR in an active state, such as while in a standing weight-bearing position, when gait outcomes are of primary concern.

### Limitations

Our findings are limited by several methodological drawbacks. One of the limitations is that cLF-rTMS was delivered to the spot that mirrored the location of iHF-rTMS and dosed on the lesioned/affected side’s rMT. This was done to reduce the number of times participants were required to undergo CMR testing by not conducting an additional hotspot identification and rMT tests. Due to the likely remodeling/remapping of the lesioned hemisphere, it is possible that during cLF-rTMS the magnetic field was not delivered to the most effective location. It is also commonly observed that the rMT of the lesioned side is higher than that of the contralesional side. In some instances, this caused the cLF-rTMS to be at or above the threshold and induce muscle contractions, although none were observed during rTMS interventions. Another limitation of our study design was that no CMR measurements were collected on the non-lesioned side to reduce the time participants underwent CMR assessments. Future investigations should consider the effects of rTMS on both hemispheres especially when using a double-cone coil it is unlikely the magnetic field is restricted to one hemisphere. Our findings indicate cLF-rTMS resulted in changes to the lesioned hemisphere that would generally be considered negative. Previous studies using rTMS delivered high-frequency stimulation to both hemispheres and greater attention may be paid to the non-lesioned side. Measuring the ipsilesional and contralesional hemispheres would also allow for the identification of CMR asymmetries. The CMR/motor control of upper and lower extremities have many differences (Kesar et al., 2018), and typical upper extremity rTMS is delivered at 90% or rMT. However, due to the increased depth of the motor cortical representations of the leg musculature additional stimulator output may be necessary to maximize the neuromodulatory effects of rTMS. Subthreshold power output in the range of 95–99% of rMT may allow for greater neuromodulation when lower extremity musculature is of interest. Future research into these areas is still necessary.

Our study advances sham controls for lower extremity rTMS studies. Currently, there are no true double-blind, sham-controlled studies examining rTMS and physical rehabilitation post-stroke. We acknowledge that our sham may not have been completely effective at blocking the induced magnetic fields by the double-cone coil, as we did not have the capability of testing the magnetic field below the coil. This may also be reflected in the fact that there were small effect sizes seen with sham stimulation for rMT and MEP_amp_. However, the effect of sham stimulation was in the opposite direction compared to iHF-rTMS and cLF-rTMS. Changes in cortical excitability have been the hallmark descriptor when describing rTMS, i.e., excitatory or inhibitory rTMS, and changes in cortical excitability were our primary outcomes. We do not believe our results were greatly affected by any possible induced magnetic field as our sham protocol randomly assigned iHF-rTMS and cLF-rTMS. Analysis of the CMR changes with sham stimulation also had no association with any clinical changes or neuroimaging variables. Future work to create* bona fide* sham conditions is still required to reach gold standard clinical trial rigor.

### Future Directions and Conclusions

Several reports in clinical populations have shown that the addition of rTMS to task-oriented training over several weeks has led to greater improvements in clinical outcomes compared to training without rTMS post-stroke (Wang et al., 2012; Kakuda et al., 2013; Chieffo et al., 2014). In motor rehabilitation, rTMS likely provides a priming effect that enhances task-oriented training and rehabilitation neuroplasticity. Certain portions of the population may respond well to neuromodulatory techniques and others may not respond at all. In the case of chronic stroke, these individual differences are further complicated by the likelihood that some people will respond more appropriately to high-frequency or low-frequency and ipsilesional or contralesional stimulation. Determining who will respond to specific rTMS treatment options before prescribing rTMS and gait rehabilitation may save time and money for patients and clinicians who are employing rTMS to enhance clinical outcomes post-stroke. It should also be reiterated that this protocol only tested the immediate effects of rTMS on the CMR as reflected by changes in MEP_amp_ and nLAT. How a single rTMS session affects task-oriented training is still unclear, especially considering that gait-rehabilitation is likely to facilitate changes within the entire neuromuscular system and not just in the cortex. Changes in cortical excitability cannot be wholly attributed to changes at the cortical level because the MEP is influenced by all structures between the cortical neuron and the actin-myosin cross-bridge. Techniques to separate cortical changes from subcortical/network communication changes may enhance our understanding of gait rehabilitation and neuromodulation for rehabilitation and better direct future research protocol design.

In conclusion, the results from this investigation seem to indicate that neither a single session of ipsilesional high-frequency nor a single session of contralesional low-frequency rTMS modulates the CMR in chronic stroke at the group level. However, we also show a range of individual responses to high-frequency and low-frequency rTMS which may suggest a need to individualize rTMS before treatment application. In our sample rTMS was well tolerated and there were no adverse events/effects and continued work integrating rTMS into motor rehabilitation may one day provide patients with chronic stroke greater clinical recovery leading to improved physical function, quality of life, and community reintegration.

## Data Availability Statement

The raw data supporting the conclusions of this article will be made available by the authors, without undue reservation.

## Ethics Statement

The studies involving human participants were reviewed and approved by Institutional Review Board, Medical University of South Carolina. The patients/participants provided their written informed consent to participate in this study. Written informed consent was obtained from the individual(s) for the publication of any potentially identifiable images or data included in this article.

## Author Contributions

JK: analyzed and interpreted data, wrote the first draft of the manuscript. EW and CC: participated in data collection, critically revised the manuscript for the intellectual and scientific content. SS, BM and LB: analyzed and interpreted data, critically revised the manuscript for the intellectual and scientific content. SK: provided conceptual framework of submitted work, critically revised the manuscript for the intellectual and scientific content. MB: provided conceptual framework of submitted work, supervised all data collection, ensured adherence to ethical guidelines, critically revised manuscript for intellectual and scientific content, and has given final approval for manuscript submission. All authors contributed to the article and approved the submitted version.

## Conflict of Interest

The authors declare that the research was conducted in the absence of any commercial or financial relationships that could be construed as a potential conflict of interest.

## Abbreviations

cLF-rTMS: contralesional low-frequency repetitive transcranial magnetic stimulation
CMR: corticomotor response
EMG: electromyography
iHF-rTMS: ipsilesional high-frequency repetitive transcranial magnetic stimulation
MEP: motor evoked potential
MEP_amp_: motor evoked potential amplitude
nLAT: normalized motor evoked potential latency
PEST: parameter estimation by sequential testing
rMT: resting motor threshold
rTMS: repetitive transcranial magnetic stimulation
sEMG: surface electromyography
TA: tibialis anterior
TMS: transcranial magnetic stimulation.
